# Primary Endoscopic Closure of Duodenal Perforation Secondary to Biliary Stent Migration: A Case Report and Review of the Literature

**DOI:** 10.1177/2324709618792031

**Published:** 2018-08-02

**Authors:** Samson Ferm, Constantine Fisher, Akil Hassam, Moshe Rubin, Sang-Hoon Kim, Syed Ahsan Hussain

**Affiliations:** 1 New York-Presbyterian Queens, Department of Internal Medicine; Flushing, NY, USA; 2 New York-Presbyterian Queens, Division of Gastroenterology; Flushing, NY, USA

**Keywords:** endoscopy, duodenum, perforation, biliary prosthesis

## Abstract

Duodenal perforation due to biliary stent migration is rare, and it often requires surgical repair; however, endoscopic closure has recently become a viable option in the appropriate patients. We present the case of a 79-year-old female who underwent biliary stent placement for a common bile duct stricture, who subsequently was found to have a duodenal wall perforation secondary to stent migration. The stent was extracted endoscopically with successful defect closure using a ConMed^®^ repositional DuraClip™. We aim to contribute to the limited body of literature that describes endoscopic repair of duodenal perforation secondary to biliary stent migration using through-the-scope endoclips.

## Introduction

The duodenum is perforated more readily than other portions of the intestine due to its comparatively thin wall. Although a number of etiologies may account for this occurrence, biliary stent migration has been reported as the cause in rare instances. Advances in endoscopic surgery have led to an increased use of endoclips to close luminal defects, yet there are few reports of the utilization of through-the-scope (TTS) endoclips to close defects caused by migrated biliary stents. We present the case of a 79-year-old female who developed a duodenal wall perforation secondary to biliary stent migration, which was extracted and closed using a ConMed^®^ repositional DuraClip™. This is the first reported use of this TTS endoclip for a defect caused by biliary stent migration.

## Case Report

A 79-year-old female with past medical history of chronic lymphocytic leukemia (in remission) presented to New York-Presbyterian Queens with complaint of chest pain radiating to the back with associated nausea.

On examination, she was afebrile with blood pressure 161/78 mm Hg. She had mild epigastric tenderness to palpation with no guarding or rigidity. Initial laboratory values revealed a white blood cell count of 7.33 K/µL, hemoglobin of 13.3 g/dL, and normal electrolytes. Cardiac enzymes were negative, and liver function tests revealed aspartate transaminase 492 U/L, alanine transaminase 493 U/L, and alkaline phosphatase 353 U/L. Electrocardiography showed no acute ischemic changes.

Abdominal ultrasound revealed a dilated common bile duct (CBD; 1 cm) with slightly dilated gallbladder without stones. Magnetic resonance cholangiopancreatography confirmed CBD dilation without stone or definite stricture. However, subsequent endoscopic retrograde cholangiopancreatography (ERCP) showed a biliary stricture at the hepatic duct bifurcation. A sphincterotomy was performed, biopsies were obtained, and 2 Advanix™ biliary stents (Boston Scientific Corporation, Natick, MA) were placed. One 7-Fr, 12-cm plastic stent was placed into the right system, and a second 10-Fr, 15-cm plastic stent went into the left system, past the stricture into the right and left hepatic ducts and extending distally into the CBD with adequate bile flow. The patient tolerated the procedure well without complications.

**Figure 1.**
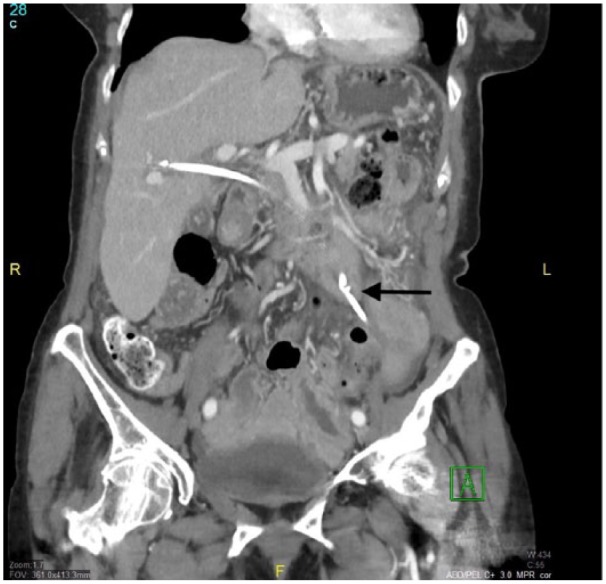
Computed tomography scan of abdomen with contrast showing distal portion of stent traversing luminal wall into peritoneal cavity (arrow).

**Figure 2.**
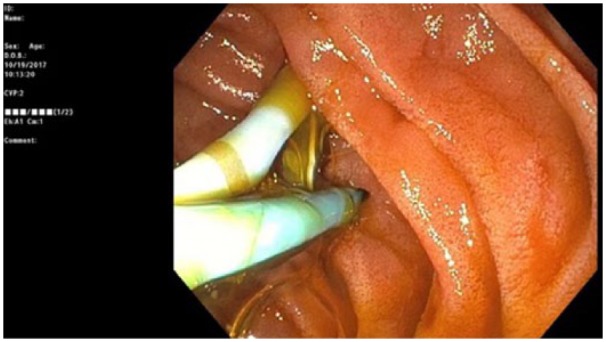
Endoscopic image showing perforation of duodenum with distal tip of biliary stent.

**Figure 3.**
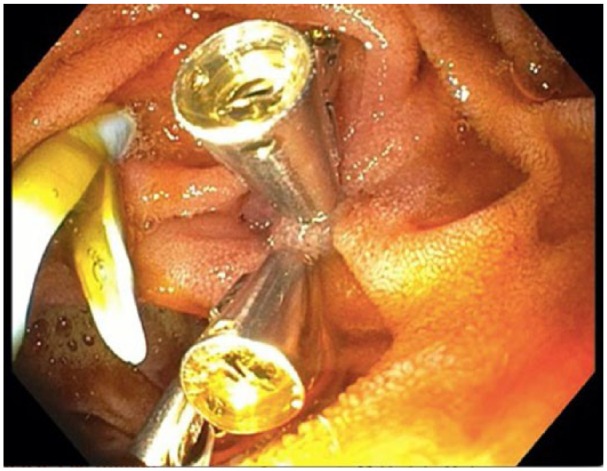
Endoscopic image showing closure of defect with 4 through-the-scope clips.

After discharge, she immediately began to experience nausea, vomiting, and abdominal pain. On examination, she was markedly tender to palpation in the left upper quadrant. Abdominal X-ray was negative for free air. Computed tomography scan revealed migration of the 7-Fr, 12-cm stent through the duodenal wall. An upper endoscopy was performed, confirming penetration of the stent through the lateral portion of the second part of the duodenum (Figure 1). The stent was then extracted using a Raptor™ grasping device (US Endoscopy, Mentor, OH) revealing a 0.5 × 0.5 cm transmural defect (Figure 2). This was closed using four 11-mm TTS endoclips (DuraClip, ConMed Corporation, Utica, NY; Figure 3).

She tolerated the procedure well, was started on levofloxacin and metronidazole, and was monitored in the hospital. She remained clinically stable and was started on clear liquid diet on post-procedure day 2. She was discharged on post-procedure day 4 and had no complications in follow-up 2 weeks after discharge.

## Discussion

Endoscopic biliary stent placement is used in the management of both benign and malignant CBD strictures. Although it is considered safe, complications such as stent migration and occlusion have been reported in approximately 5% and 10% to 30% of patients, respectively.^1,2^

Duodenal perforation secondary to stent migration is well documented in the literature and occurs in less than 1% of patients.^3-6^ The decision to treat medically, surgically, or endoscopically depends on the site of injury, size and type of perforation, and operator experience. Conservative management is appropriate for stable patients with small defects in the absence of large fluid collections and/or peritonitis.^7,8^

With regard to patients who do not respond to conservative management, surgery or endoscopy is required, with the former more commonly performed.^9^ The mortality associated with surgery after failed conservative management can be high, with reports between 13% and 37.5%.^10,11^

Endoscopic management of defects due to biliary stent migration has been reported with over-the-scope clips (OTSC), which confer the advantage of deeper tissue closure as compared with TTS clips. Javia et al endoscopically repaired a duodenocolic fistula using an OTSC.^12^ Kriss et al repaired a duodenal perforation with an OTSC in a liver transplant recipient.^13^

The use of TTS clips in the management of duodenal defects due to biliary stent migration is limited due to concerns about closure strength and jaw opening width.^14^ In review of the literature, only a few case reports highlight this technique. Rosés et al described the use of a TTS hemoclip placement (Olympus America, Melville, NY) for a duodenal perforation due to a 10-Fr, 15-cm plastic biliary stent.^15^ Similarly, Sanchez-Ocana et al performed a TTS clip closure of a duodenocolic fistula in the setting of biliary stent migration.^16^

**Figure 4.**
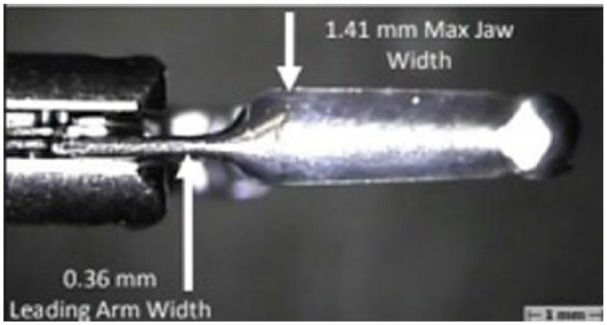
ConMed repositional hemostasis clip (Duraclip) showing thin leading arm and jaw width.

This is the first case report of a closure of a duodenal defect secondary to biliary stent migration using the ConMed repositional hemostasis clip (Duraclip). The Duraclip was chosen in this instance for its thin leading arm, which we surmised would allow for easier visualization of the defect (Figure 4). Despite the thin arm, our case demonstrates that this clip has enough retention strength to close this type of mucosal defect within the gastrointestinal tract. A complex surgical procedure was avoided using this approach. Our case highlights the first reported experience using a novel therapeutic accessory for duodenal perforation caused by biliary stent migration specifically. Additionally, we add to the paucity of literature that describes TTS clip management of this uncommon gastrointestinal pathology. Larger studies are required to compare traditional surgical techniques with the outcomes of endoscopic closure using endoclips.
